# Supplementary material for: Efficient Pervaporation for Ethanol Dehydration: Ultrasonic Spraying Preparation of Polyvinyl Alcohol (PVA)/Ti3C2Tx Nanosheet Mixed Matrix Membranes
Source: Membranes (Basel). 2023 Apr 13;13(4):430. doi: 10.3390/membranes13040430 (PMC10146547; doi:10.3390/membranes13040430)
Supplement: Supplementary file 1 [file membranes-13-00430-s001.zip › membranes-2270564-supplementary.pdf]

Efficient pervaporation for ethanol dehydration: Ultrasonic spraying preparation of polyvinyl alcohol (PVA)/ $Ti_3C_2T_x$ nanosheet mixed matrix membranes

Huijuan Tong, Qiao Liu*, Nong Xu*, Qing Wang, Long Fan, Qiang Dong, Aiqin Ding

School of Energy, Materials and Chemical Engineering, Hefei University, Hefei 230601, P. R. China

* Corresponding authors, *E-mail addresses*: liuqiao@hfuu.edu.cn (Q. Liu), xunong@hfuu.edu.cn (N. Xu)

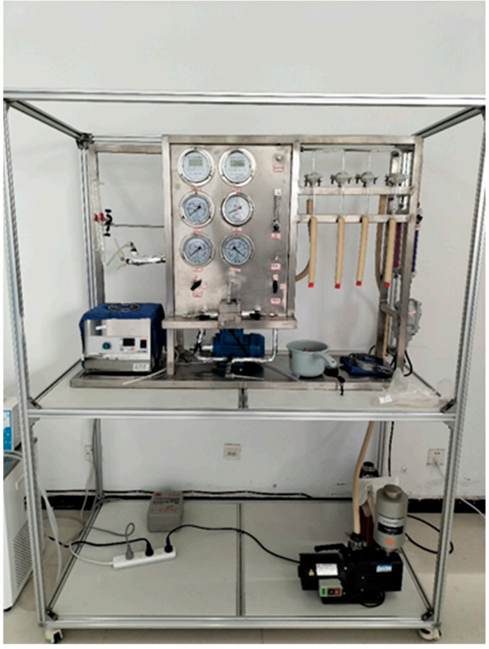

Figure S1. Photograph of the homemade PV device

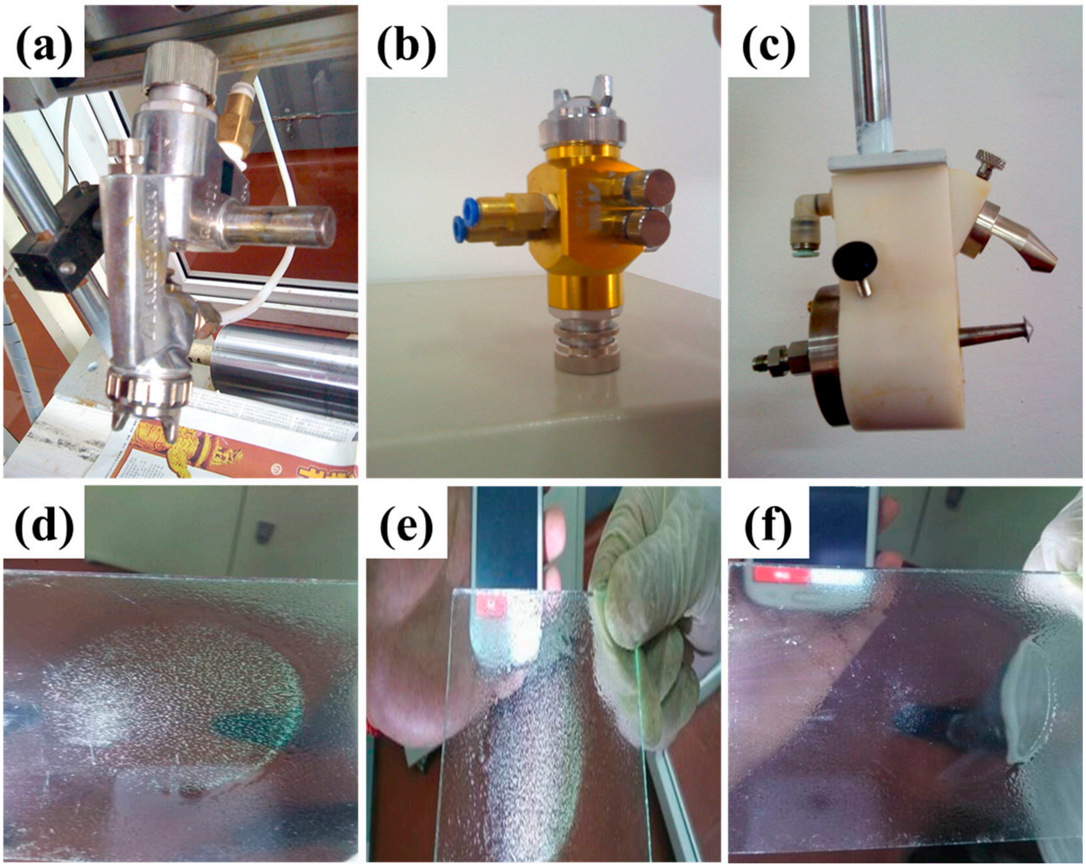

Figure S2. Two different types of gas dynamic spray nozzles, (a) WA-101 and (b) TOF-30-1.5 (Anest-Iwata. Co. Japan), were tried in our spraying processes. (c) ultrasonic spraying nozzle (FSW-6001-L, Funsonic Co. Ltd. China) used in the preparation of the membranes. (d), (e) and (f) were the sprayed fine droplets of the PVA-based solutions on the glass plates after the solutions were puffed out from the WA-101, TOF-30-1.5 and FSW-6001-L, respectively.

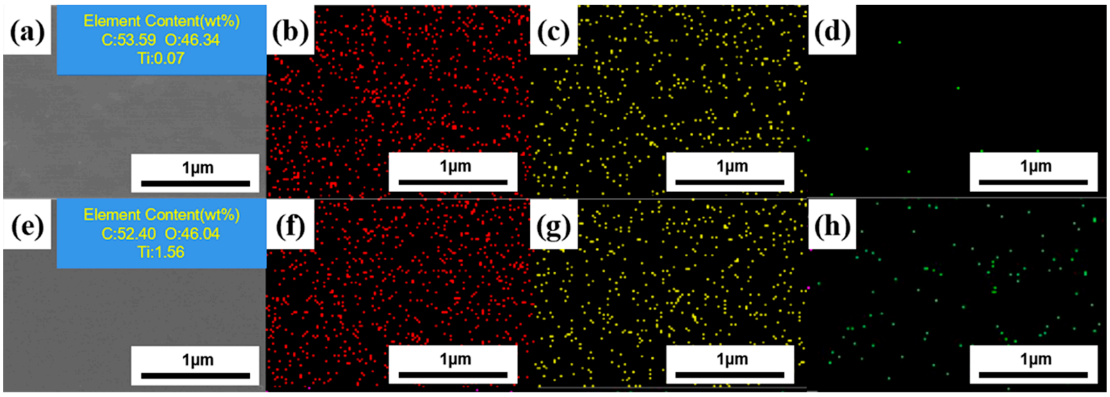

Figure S3. (a) and (e) SEM images, EDX mapping images of (b) and (f) C element, (c) and (g) O element, (d) and (h) Ti element in the PVA and PGM-0 membranes' top-surface, respectively.

Table S1. The PV data for all the prepared composite membranes in this work

Samples	Water flux ($kg\cdot m^{-2}\cdot h^{-1}$)	Separation factor
PVA	0.26±0.03	125.4±36.9
PG	0.18±0.04	133.6±28.4
PGM-0	1.21±0.02	1126.8±33.7
PGM-1	1.05±0.03	1108.4±31.2
PGM-2	0.95±0.02	874.3±29.6
PGM-3	0.92±0.03	732.8±30.7
PGM-4	0.88±0.04	687.5±28.4
PGM-5	1.08±0.03	1084.9±35.4
PGM-6	0.96±0.04	823.4±29.8
PGM-7	1.10±0.02	1045.8±32.4
PGM-8	1.07±0.03	1102.4±36.7
PGM-9	0.94±0.04	976.4±36.8
PGM-10	0.82±0.03	875.3±37.5

Table S2. Comparison of the PV performance in this work with others

Membrane	Symbol	Temp. °C	Feed (ethanol wt %)	Water flux ($kg \cdot m^{-2} \cdot h^{-1}$)	Separation factor	Ref.
PVA	◭	40	90	0.280	104	[1]
PVA/ZIF-90	⊗	30	90	0.268	1379	[2]
PVA/SiO_2	◆	40	85	0.145	1026	[3]
PVA/GO	■	45	95	0.074	4281	[4]
CS/siloxane	⬣	20	90	0.47	2182	[5]
CS	●	40	96	0.004	2208	[6]
SA/4A	△	30	90	0.111	1866	[7]
SA/PVP/PWA	◁	27	90	0.100	1250	[8]
HA/SA/PAN	⬟	80	90	0.90	1130	[9]
PAA/PEI/PAN	⊞	50	85	1.00	100	[10]
PAA/PEI/PES	▷	40	85	0.47	350	[11]
PGM-0	★	30	95	1.21	1127	This work

References

[1] Yeom, C. K.; Lee, K, H. Characterization of sodium alginate and poly(vinyl alcohol) blend membranes in pervaporation separation. *J. Appl. Polym. Sci*, **2001**, *67(5)*, 949–959.

[2] Wei, Z.; Liu, Q.; Wu, C.; Wang, H.; Wang, H. Viscosity-driven in situ self-assembly strategy to fabricate cross-linked ZIF-90/PVA hybrid membranes for ethanol dehydration via pervaporation. *Sep. Purif. Technol.* **2018**, *201*, 256–267.

[3] Xia, L.; Li, C.; Wang, Y. In-situ crosslinked PVA/organosilica hybrid membranes for pervaporation separations. *J. Membr. Sci*, **2016**, *498*, 263–275.

[4] Cheng, X.; Cai, W.; Chen, X.; Shi, Z.; Li, J. Preparation of graphene oxide/poly(vinyl alcohol) composite membrane and pervaporation performance for ethanol dehydration. *RSC Adv*, **2019**, *9(27)*, 15457–15465.

[5] Lin, Y.; Ho, J. C.; Lin, K. Y. A.; Tung, K. L.; Chung, T. W.; Lee, C. C. A drying-free and one-step process for the preparation of siloxane/CS mixed-matrix membranes with outstanding ethanol dehydration performances. *Sep. Purif. Technol*, **2019**, *221*, 325–330.

[6] Uragami, T.; Matsugi, H.; Miyata, A. T. Pervaporation characteristics of organic-inorganic hybrid membranes composed of poly(vinyl alcohol-co-acrylic acid) and tetraethoxysilane for water/ethanol separation. *Macromolecules*, **2005**, *38(20)*, 8440–8446.

[7] Zhao, J.; Fang, C.; Zhu, Y.; He, G.; Pan, F.; Jiang, Z.; Zhang, P.; Cao, X.; Wang, B. Manipulating the interfacial interactions of composite membranes via a

mussel-inspired approach for enhanced separation selectivity. *J. Mater. Chem. A*, **2015**, *3(39)*, 19980–19988.

[8] Magalad, V. T.; Gokavi, G. S.; Ranganathaiah, C.;. Burshe, M. H.; Han, C.; Dionysiou, D. D.; Nadagouda, M. N.; Aminabhavi, T. M. Polymeric blend nanocomposite membranes for ethanol dehydration-effect of morphology and membrane-solvent interactions. *J. Membr. Sci*, **2013**, *430*, 321–329.

[9] Gao, C.; Zhang, M.; Ding, J.; Pan, F.; Jiang, Z.; Li, Y.; Zhao, J. Pervaporation dehydration of ethanol by hyaluronic acid/sodium alginate two-active-layer composite membranes. *Carbohydr. Polym*, **2014**, *99*, 158–165.

[10] Zhang, G.; Song, X.; Ji, S.; Wang, N.; Liu, Z. Self-assembly of inner skin hollow fiber polyelectrolyte multilayer membranes by a dynamic negative pressure layer-by-layer technique. *J. Membr. Sci*, **2008**, *325(1)*, 109–116.

[11] Zhang, G.; Gu, W.; Ji, S.; Liu, Z.; Peng, Y.; Wang, Z. Preparation of polyelectrolyte multilayer membranes by dynamic layer-by-layer process for pervaporation separation of alcohol/water mixtures. *J. Membr. Sci*, **2006**, *280(1-2)*, 727–733.
